# Mtb-Timer: a fluorescent reporter to visualize *Mycobacterium tuberculosis* replication and antibiotic responses

**DOI:** 10.1128/msystems.01796-25

**Published:** 2026-05-19

**Authors:** Víctor Campo-Pérez, Chak Hon Luk, Laure Botella, Julien Vaubourgeix, Maximiliano G. Gutierrez

**Affiliations:** 1 Host-Pathogen Interactions in Tuberculosis Laboratory, The Francis Crick Institute 376570, London, United Kingdom; 2 Institut de Recherche en Santé Digestive, Université de Toulouse, INSERM, INRAE, ENVT, UPS 27091, Toulouse, France; Rice University, Houston, Texas, USA

**Keywords:** *Mycobacterium tuberculosis*, single cell, replication, macrophages

## Abstract

Despite the importance of monitoring *Mycobacterium tuberculosis* (Mtb) replication and antibiotic responses at the single-cell level, available tools to accurately monitor single Mtb replication are limited. Here, we report the development of Mtb-Timer, a fluorescent reporter in Mtb that exploits the intrinsic green-to-red photoconversion of the Timer protein as a proxy for Mtb replication. We generated an Mtb H37Rv strain expressing Timer (Timer^Mtb^) and monitored *in vitro* Mtb replication at the single-cell level by imaging and flow cytometry. Moreover, we show that exposure to the first-line anti-tuberculosis antibiotics isoniazid, rifampicin, ethambutol, and pyrazinamide resulted in a dose-dependent green-to-red photoconversion by imaging and flow cytometry. When we tested Mtb-Timer *in cellulo* using primary human monocyte-derived macrophages, we observed that intracellular Mtb populations displayed a striking phenotypic heterogeneity. Single-cell Mtb-Timer green-to-red ratios analyzed by both fixed and live imaging revealed intracellular Mtb populations with high green-to-red ratios. In contrast, treatment with antibiotics showed a decrease in green-to-red ratios in a dose-dependent manner by imaging and flow cytometry. Collectively, these results establish Mtb-Timer as a robust tool for monitoring Mtb replication and antibiotic responses, both *in vitro* and *in cellulo*, by imaging and flow cytometry.

**IMPORTANCE:**

Tuberculosis treatment is challenging in part because *Mycobacterium tuberculosis* (Mtb) populations are phenotypically heterogeneous. Within the same infection, bacterial cells can exist in different physiological states, from actively replicating to slow-growing or non-replicating forms. To better study these processes, we developed and validated Mtb-Timer, a fluorescent reporter that enables visualization of bacterial growth dynamics. The system is based on a fluorescent protein that changes color over time: newly synthesized protein emits green fluorescence (GFP) and gradually shifts to red (DsRed) as it matures. The green-to-red fluorescence ratio, therefore, reflects bacterial replication activity. Using microscopy and flow cytometry in both *in vitro* cultures and infected macrophages (*in cellulo*), Mtb-Timer enables the quantification of distinct bacterial subpopulations and the real-time monitoring of the green-to-red fluorescence ratio and bacterial burden during treatment with first-line antibiotics.

## INTRODUCTION

Studying the replication dynamics of *Mycobacterium tuberculosis* (Mtb) during infection at the single-cell resolution remains a major challenge in tuberculosis research (1). The capacity of Mtb to switch between replicating and non-replicating states underlies its remarkable adaptation to the host and its tolerance to antibiotic treatment (2). Capturing these states in real time, particularly at the single-cell level, requires molecular tools that can report bacterial replication status within complex and dynamic host environments (3). Fluorescent reporters have become valuable tools for investigating the physiological heterogeneity of Mtb during infection. Among these, dual-fluorescent reporter systems have been developed in which one fluorophore is expressed constitutively, and a second fluorophore is placed under the control of a riboswitch or a promoter-based reporter that responds to defined environmental or stress signals experienced during infection. Using this strategy, reporters responsive to conditions such as hypoxia, nitric oxide, and phagosome maturation have been developed, enabling the simultaneous assessment of bacterial presence and stress responses at the single-bacterium level within infected cells and tissues (4). These reporter strains have subsequently been applied to study host-pathogen interactions, revealing that Mtb replication and physiological state vary across macrophage populations in the lung and are linked to host cell metabolism and lineage, highlighting the heterogeneity of intracellular niches encountered during infection (5, 6). Importantly, they have been applied in *in vivo* drug studies, linking bacterial drug susceptibility to host cell phenotype (7) and to the spatial distribution of bacteria within granulomas (8). Similarly, inducible fluorescent reporter strains offer a powerful complementary approach for identifying bacteria that are actively transcribing at desired time points or enabling expression to be turned on or off (9). These systems use tightly regulated promoters to drive the expression of fluorescent proteins only upon addition of an exogenous inducer (typically tetracycline, doxycycline, or theophylline), allowing selective labeling of metabolically active bacilli at defined stages of an assay. Such reporters have been successfully applied to study single-cell replication dynamics *in vitro* by fluorescence dilution (10) and to distinguish viable and transcriptionally active bacteria in macrophages (11).

By revealing how Mtb adapts to distinct microenvironments and host immune pressures, these dual reporters provide correlative insights into the factors that influence treatment efficacy and bacterial persistence. More recently, bioluminescence-based assays have been developed to monitor Mtb responses to antibiotics in real time within host macrophages. These assays incorporate host immune stresses to more accurately reflect the intracellular environment and can capture the dynamic induction of phenotypic drug tolerance, allowing quantification of how bacterial metabolism and survival change in response to both antibiotics and host-mediated pressures (12). By providing a real-time, non-destructive readout, these approaches complement fluorescence-based reporters and offer a scalable platform to study host-pathogen-drug interactions at the single-cell level.

Building on these approaches, fluorescent timer proteins provide an additional layer of temporal resolution, complementing traditional Mtb dual reporters, which often exhibit a lag between gene expression and detectable fluorescence, meaning bacterial division is not always accurately reflected. The use of time-resolved fluorescent reporters originates from the development of the Timer protein, a derivative of DsRed, which undergoes a spontaneous and irreversible green-to-red emission shift as it matures over time (13). Newly synthesized Timer molecules emit green fluorescence, which gradually shifts to red due to oxidation-driven conformational changes in the protein. As a result, older molecules appear red, allowing the green-to-red fluorescence ratio to serve as an intrinsic molecular clock for protein age. In bacteria, the first application of the fluorescent Timer was reported in *Salmonella*, where it was used to monitor replication dynamics during infection and to distinguish actively replicating from growth-arrested subpopulations within host tissues (14). This pioneering work demonstrated the potential of Timer-based reporters to reveal phenotypic heterogeneity in pathogenic bacteria. Subsequent studies extended and validated this approach in other species (15, 16).

Applying an optimized Timer reporter plasmid to Mtb (Timer^Mtb^) represents a valuable opportunity, given the highly complex and dynamic intracellular lifestyle of the bacilli (17). In the host, Mtb populations display a remarkable and unique heterogeneity in replication rates, metabolic activities, and responses to host-derived stress (18). Subpopulations of intracellular Mtb are exposed to distinct microenvironments that profoundly influence their replication dynamics, access to nutrients, and susceptibility to antibiotics, revealing spatial and temporal heterogeneity even within single infected cells (19, 20). Adding more complexity, Mtb can reside within different intracellular environments with variable degrees of residence in membrane-bound compartments or the cytosol (21).

The use of a Timer reporter in Mtb can also significantly contribute to *in vitro* drug-screening approaches when combined with established agarose pad microscopy methods (22, 23), which are often limited to morphological measurements (24). A Timer approach in Mtb could provide a functional readout that distinguishes growing from non-growing subpopulations by measuring the green-to-red fluorescence ratios. This approach could potentially enable the detection of drug-tolerant bacteria that may be overlooked by growth-based assays and enhance single-bacterium phenotyping during antibiotic exposure. Moreover, Timer also enables the detection of metabolically active and inactive bacteria by flow cytometry (25). In infected host cells, the distinct fluorescence states can allow rapid quantification of subpopulations after antibiotic treatment, as well as the identification of infected host cells enriched in predominantly green or red Mtb.

Here, we report the development of Mtb-Timer to quantitatively monitor and analyze the spatiotemporal dynamics of Mtb replication at the single-cell level. We found that the ratiometric measure of Mtb-Timer enabled real-time discrimination between replicating and non-replicating bacteria at both single-cell and population levels. Importantly, we show that Mtb subpopulations after antibiotic treatment can be monitored using Mtb-Timer by both imaging and flow cytometry.

## RESULTS

### Mtb-Timer reveals single-bacterium phenotypic heterogeneity *in vitro*

**Fig 1:**
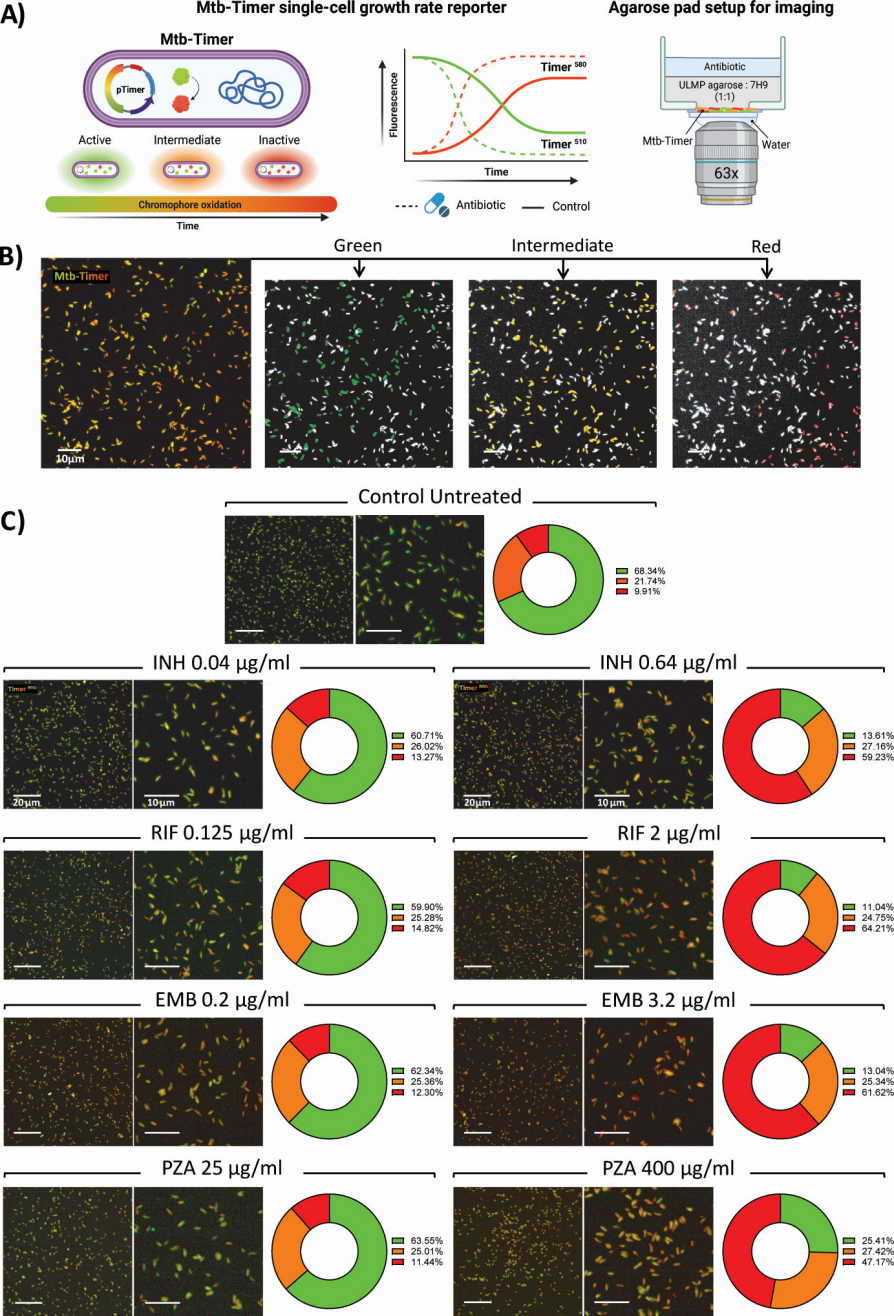
Single-cell analysis of Mtb-Timer reporter strain under antibiotic exposure. (**A**) Illustration of the principle of the Mtb-Timer strain, where fluorescence gradually shifts from green to red. Graph showing that antibiotic treatment accelerates this green-to-red fluorescence shift. A final representation depicts the agarose pad imaging setup: bacteria positioned at the bottom of the well, overlaid with agarose containing 7H9 and antibiotic. Created with Bio Render. (**B**) Representative image showing Mtb-Timer phenotypic heterogeneity within agarose pads and segmentation of individual bacteria into actively replicating (green), intermediate (orange), or non-replicating (red) subpopulations based on their single-bacterium green-to-red fluorescence ratio. (**C**) Representative images of Mtb-Timer grown on agarose pads and exposed to the lowest (left) and highest (right) concentrations of the first-line antituberculosis drugs: isoniazid (INH), rifampicin (RIF), ethambutol (EMB), and pyrazinamide (PZA) for 24 h. Quantifications show the percentage of area occupied by each replication phenotype: green (active), orange (intermediate), and red (inactive) relative to the total bacterial area in each condition. Values correspond to the mean of three independent biological replicates.

To monitor Mtb replication at the single-cell level, we generated an Mtb H37Rv strain expressing an Mtb codon-optimized Timer^Mtb^ as previously shown in other bacteria (26) (Fig. 1A). Timer^Mtb^ undergoes a spontaneous, time-dependent spectral shift from green-to-red emission, providing a ratiometric indicator of protein age. To analyze the behavior of the Timer^Mtb^ at the single-cell level *in vitro*, Mtb-Timer cultures were immobilized on agarose pads placed on 96-well plates and imaged (see Materials and Methods). Bacteria were segmented and classified based on Mtb-Timer fluorescence ratio into green (metabolically active ≥0.9), orange (0.7 < intermediate < 0.9), or red (inactive ≤0.7) subpopulations (Fig. 1B; Fig. S1). This approach enabled quantitative analysis of the area occupied by each fluorescent subpopulation, providing a direct measure of bacterial metabolic states within the same culture (Fig. 1C). The single-cell agarose pad approach revealed a striking heterogeneity among individual bacilli, allowing the identification of subpopulations with distinct growth and metabolic profiles. Under low-concentration antibiotic (1/16 EC_99_), the subpopulation structure within the agarose pad remained dominated by actively replicating cells (~60% bacteria), with proportions of intermediate (~25% bacteria) and non-replicating (~15% bacteria) cells, comparable to the values observed in the untreated control. In contrast, when exposed to high antibiotic concentrations, this distribution shifted markedly, with the non-replicating subpopulation becoming dominant (~50%–60% bacteria). Notably, even under these high-stress conditions imposed by the presence of antibiotics, a detectable fraction of metabolically active bacteria persisted (~10%–20%). These results demonstrate that agarose pad imaging, combined with Mtb-Timer, provides a robust platform to visualize and quantify single-bacterium phenotypic diversity, offering valuable insight into Mtb metabolic heterogeneity and population dynamics *in vitro*.

### Mtb-Timer accurately captures antibiotic-induced replication arrest *in vitro*

**Fig 2:**
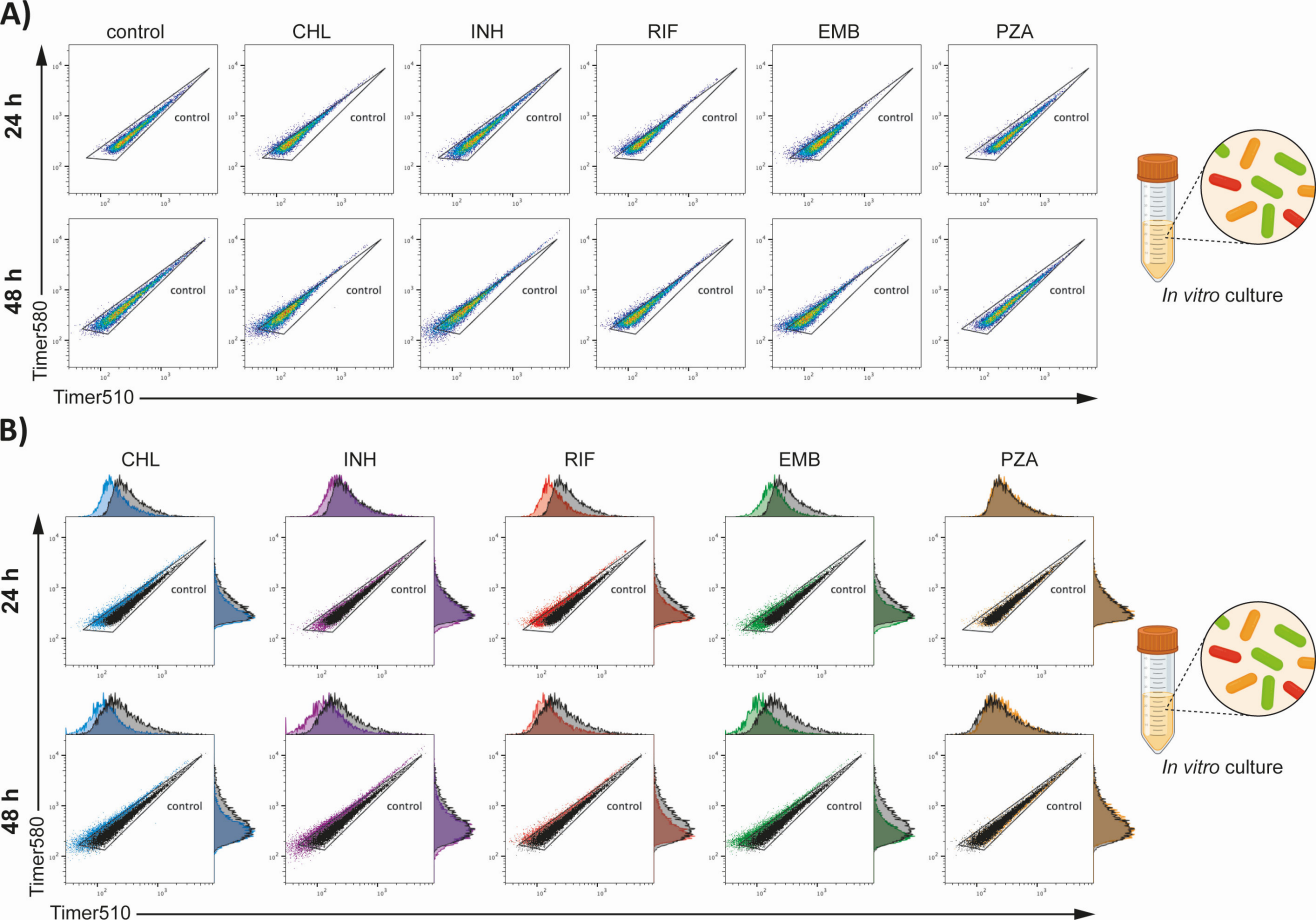
Flow cytometry analysis of Mtb-Timer *in vitro*. (**A**) Flow cytometry dot plots of *in vitro* Mtb-Timer cultures showing Timer580 vs Timer510 at 24 and 48 h after the supplementation of antibiotics. Plots illustrate the positioning and spread of bacterial populations within the predefined Timer gate for control and treated conditions. (**B**) Flow cytometry dot plots of *in vitro* Mtb showing Timer580 vs Timer510 at 24 and 48 h after the supplementation of antibiotics; plot shows treatment with CHL, 3.2 µg/mL (blue); INH, 0.64 µg/mL (purple); RIF, 2 µg/mL (red); EMB, 3.2 µg/mL (green); and PZA, 400 µg/mL (orange) overlaying with control (black). Marginal histograms are included to highlight distribution shifts. Data shown are representative of one of three independent biological replicates, all of which displayed consistent patterns and trends.

As a proof of concept, we tested the robustness of the Mtb-Timer reporter by analyzing the functional readout of Mtb-Timer in response to antibiotic treatment. We supplemented the log-phase Mtb *in vitro* culture with four first-line anti-TB antibiotics (isoniazid [INH], rifampicin [RIF], ethambutol [EMB], and pyrazinamide [PZA]) and the bacteriostatic antibiotic chloramphenicol (CHL) as a positive control. Upon addition of antibiotics *in vitro*, we observed growth arrest of Mtb-Timer treated with INH, RIF, EMB, and CHL. There was no impact on replication after PZA treatment, in agreement with the requirement of acidic pH for PZA activity (27). Using flow cytometry analysis, we detected that *in vitro* Mtb-Timer showed a lower green intensity and lower green-to-red ratio upon treatment with CHL, INH, RIF, and EMB, but not with PZA at both 24 and 48 h after treatment (Fig. 2). These observations correlate with the *in vitro* growth curve of Mtb (Fig. S2) and with the values obtained in the agarose pad procedure (Fig. 1C), demonstrating the functionality of Mtb-Timer readout as a measurement of bacterial replication and responses to antibiotics *in vitro*.

### Mtb-Timer reveals heterogeneous replication states at the single-cell level *in cellulo*

**Fig 3:**
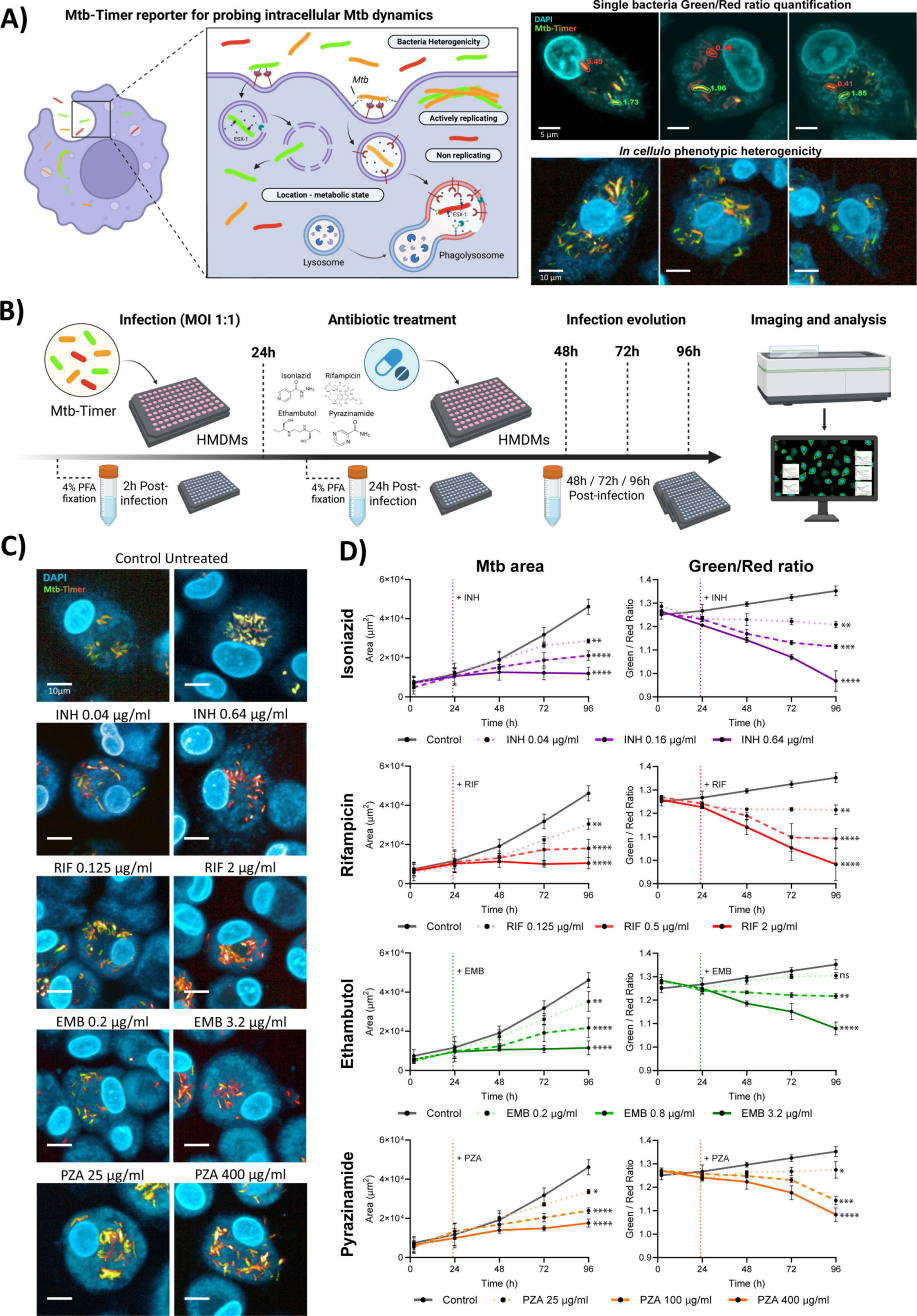
Mtb-Timer reveals a correlation between replication and fluorescent ratio. (**A**) Illustration of the advantages of the Mtb-Timer reporter for studying intracellular Mtb dynamics and distinguishing actively replicating from non-replicating bacteria. Representative images of human monocyte-derived macrophages (HMDMs) infected with Mtb-Timer are shown, highlighting individual bacteria with their corresponding green-to-red fluorescence ratio values and revealing the heterogeneity of intracellular bacterial states within single macrophages. (**B**) Schematic representation of the experimental workflow: infection of HMDMs with Mtb-Timer for 24 h prior to antibiotic addition, fixation of plates at 2, 24, 48, 72, and 96 h post-infection, followed by imaging and analysis. Created with BioRender. (**C**) Representative images of HMDMs at 96 h post-infection, treated with the lowest and highest concentrations of each antibiotic. (**D**) Quantification of intracellular Mtb area and green-to-red fluorescence ratio in HMDMs infected with Mtb-Timer (MOI 1:1). Data are expressed as mean ± standard deviation from three independent biological replicates. Statistical analysis was performed using a two-way ANOVA followed by Dunnett’s multiple comparisons test, with each antibiotic concentration compared to the untreated control condition. ns, not significant; *P ≤ 0.05; **P ≤ 0.01; ***P ≤ 0.001; and ****P ≤ 0.0001.

During infection, a substantial fraction of Mtb resides inside host cells, primarily macrophages (18, 28). We then aimed to study the behavior of Mtb-Timer during infection of human primary macrophages. Under baseline conditions, the green-to-red fluorescence transition occurred gradually. In contrast, the diverse intracellular microenvironments encountered within macrophages affected Mtb metabolic activity, and these shifts were accurately captured by the Mtb-Timer reporter (Fig. 3A). When localized within human macrophages, Mtb-Timer allowed the visualization of heterogeneous intracellular bacterial populations showing distinct fluorescence profiles. Representative confocal images revealed green fluorescent bacilli corresponding to actively replicating bacteria, orange intermediates reflecting slowed growth, and red fluorescent bacilli representing non-replicating or metabolically inactive populations (Fig. 3A). These findings demonstrate that Mtb-Timer discriminated between replicating and non-replicating Mtb at the single-cell level. We then analyzed the relationship between Mtb replication monitored by Mtb-Timer in infected human macrophages and the effect of antibiotics against intracellular Mtb. Human primary monocyte-derived macrophages were infected with Mtb-Timer at a multiplicity of infection (MOI) of 1:1 and fixed at 2, 24, 48, 72, and 96 h after infection. The first-line anti-TB antibiotics, INH, RIF, EMB, and PZA, were added 24 h post-infection, reflecting low (1/16 EC_99_), medium (1/4 EC_99_), and high (EC_99_) concentrations (Fig. 3B). At 96 h post-infection, we observed that low-dose treatments displayed heterogeneous populations with mostly actively replicating (green) and slow-replicating (orange) bacilli, whereas high-dose treatments showed predominantly non-replicating (red) bacilli. Importantly, even at the highest drug concentrations, some individual intracellular bacilli retained replication activity (green-orange fluorescence), suggesting bacterial survival despite strong population-level growth inhibition (Fig. 3C). Quantification of intracellular Mtb area and the green-to-red fluorescence ratio revealed a clear correlation between growth and Mtb-Timer signal (Fig. 3D). In untreated controls, bacterial area increased over time, accompanied by a predominance of green fluorescence, consistent with metabolically active and replicating bacilli. In the presence of antibiotics, even at the lowest concentrations, intracellular Mtb replication was reduced compared to untreated controls, and the green-to-red ratio was markedly lower (Fig. 3D). High antibiotic concentrations fully inhibited Mtb replication, in agreement with low green-to-red ratios and a predominance of red fluorescence (Fig. 3D). These findings demonstrate that Mtb-Timer allows quantitative assessment of both intracellular Mtb replication and the heterogeneity of metabolic states within macrophages, as well as providing a sensitive, single-cell readout of antibiotic efficacy.

### Mtb-Timer captures replication rates and antibiotic-induced shifts *in cellulo*

**Fig 4:**
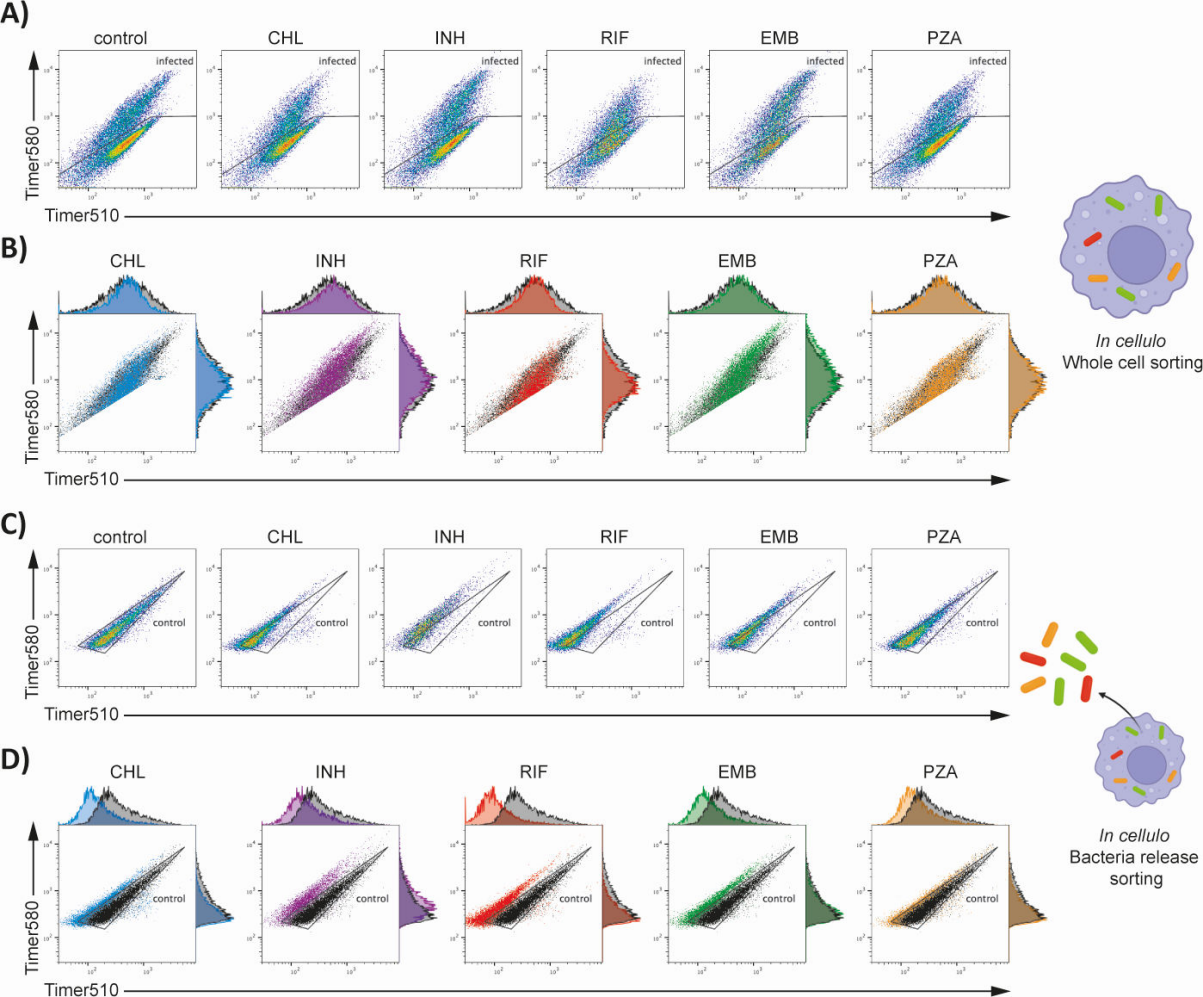
Flow cytometry analysis of Mtb-Timer *in cellulo*. (**A**) Flow cytometry dot plots of THP-1 cells showing Timer580 vs Timer510 at 72 h after infection. (**B**) Flow cytometry dot plots of infected THP-1 cells showing Timer580 vs Timer510 at 72 h after infection; plot shows treatment with CHL, 10 µg/mL (blue); INH, 0.64 µg/mL (purple); RIF, 2 µg/mL (red); EMB, 3.2 µg/mL (green); and PZA, 400 µg/mL (orange) overlaying with control (black). (**C**) Flow cytometry dot plots of Mtb harvested from THP-1 cells showing Timer580 vs Timer510 at 72 h after infection. (**D**) Flow cytometry dot plots of Mtb harvested from THP-1 cells showing Timer580 vs Timer510 at 72 h after infection; plot shows treatment with CHL, 10 µg/mL (blue); INH, 0.64 µg/mL (purple); RIF, 2 µg/mL (red); EMB, 3.2 µg/mL (green); and PZA, 400 µg/mL (orange) overlaying with control (black).

To demonstrate the capacity of Mtb-Timer to monitor Mtb replication rates as well as responses to antibiotic treatment *in cellulo*, we analyzed Mtb-Timer-infected THP-1 human macrophages. Differentiated THP-1 macrophages were infected with Mtb-Timer at an MOI of 1 and treated with first-line antibiotics and CHL (as a positive control) at 24 h post-infection or left untreated, and cells were harvested at 72 h post-infection. Using flow cytometry analysis, we observed a substantial overlap between untreated infected cells (control) and cells treated with first-line anti-TB antibiotics, particularly in EMB- and PZA-treated macrophages. However, despite this overall similarity, a subtle shift in the Mtb-Timer profiles of infected THP-1 cells was detectable in response to CHL, INH, and RIF antibiotic treatments (Fig. 4A and B). Notably, flow cytometry analysis of infected THP-1 cells analyzed the collective response against antibiotics of all intracellular Mtb within each infected cell, where Timer profile shift of individual bacterium is masked by the total fluorescence of an infected cell. To further investigate the Mtb responses against antibiotics at single-cell level, we lysed the infected THP-1 cells and analyzed the Timer profiles of intracellular Mtb by flow cytometry. We observed the reduction of Timer-green intensity and green-to-red ratio among conditions treated with INH, RIF, EMB, and CHL, as well as a limited reduction with PZA (Fig. 4C and D), in agreement with our imaging data (Fig. 3C). Altogether, our data show the capacity of Mtb-Timer in capturing Mtb heterogeneity and antibiotic responses at single-macrophage and single-Mtb levels using fluorescence microscopy and flow cytometry.

### Live monitoring of intracellular Mtb-Timer replication and antibiotic responses

**Fig 5:**
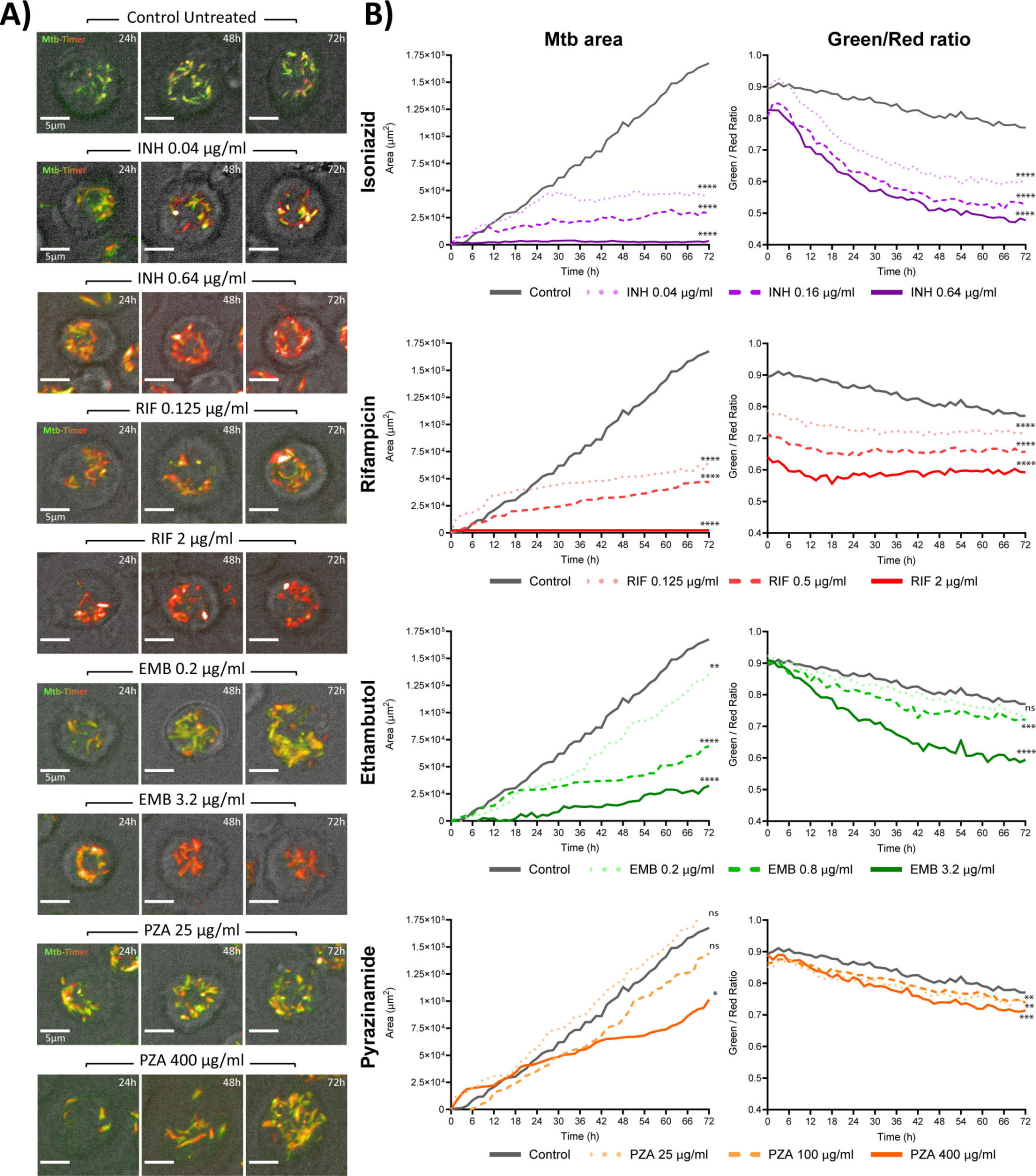
Antibiotic-dependent modulation of intracellular Mtb-Timer growth and fluorescence dynamics in live-infected human monocyte-derived macrophages (HMDMs). (**A**) Representative live-cell images of the same HMDM infected with Mtb-Timer at 24, 48, and 72 h post-infection. Images are shown for low and high concentrations of INH, RIF, EMB, and PZA. Bacteria in macrophages exposed to higher antibiotic concentrations appear predominantly red, whereas those under lower concentrations display mixed green-orange fluorescence, reflecting differences in bacterial physiological state. (**B**) Quantification of intracellular Mtb growth (bacterial area) in untreated or treated cells with low, medium, or high concentrations of each antibiotic, together with the corresponding green-to-red fluorescence ratio over 72 h of live infection. Graphs are representative of one out of three independent biological replicates. Statistical analysis was performed using a two-way ANOVA, followed by Dunnett’s multiple comparisons test, in which each antibiotic concentration was compared with the untreated control condition at the corresponding time points. ns, not significant; *P ≤ 0.05; **P ≤ 0.01; ***P ≤ 0.001; and ****P ≤ 0.0001.

We next sought to test if Mtb-Timer captures the dynamic behavior of intracellular Mtb and the responses to antibiotic treatment. For that, primary human macrophages were infected with Mtb-Timer and analyzed by live cell imaging over 72 h under low (1/16 EC_99_), medium (1/4 EC_99_), and high (EC_99_) concentrations of INH, RIF, EMB, or PZA. Quantitative analysis of bacterial area and green-to-red fluorescence ratio revealed that Mtb intracellular replication strongly correlated with Mtb-Timer signal: actively replicating bacteria maintained a higher green-to-red ratio, whereas growth inhibition corresponded to a lower ratio (Fig. 5A). At high antibiotic concentrations, reduced replication was observed for both INH and RIF treatments (Fig. 5A). In contrast, slight growth was detected under high EMB treatment, and more pronounced growth persisted under PZA, reflecting its higher activity *in vivo* compared to *in cellulo*. Representative images at 24, 48, and 72 h post-infection highlight the temporal dynamics of bacterial populations within individual macrophages, showing a progressive accumulation of red-fluorescent, non-replicating bacteria under effective antibiotic treatment, while metabolically active bacilli remained at lower concentration of antibiotics (Fig. 5B). Altogether, these data show that Mtb-Timer allows for monitoring intracellular Mtb replication and responses to antibiotics.

## DISCUSSION

Here, we report the development of a fluorescent Timer reporter in Mtb (Mtb-Timer) that represents a methodological advance to study Mtb biology at the single-cell level. Timer proteins have been applied to other bacteria but have not yet been optimized for Mtb (Timer^Mtb^). We show that Mtb-Timer works *in vitro* and in infected macrophages and informs responses to antibiotics. Mtb is characterized by an extraordinary capacity to enter non-replicating or slow-replicating states in response to environmental stress, including nutrient limitation, hypoxia, host immune pressure, or antibiotic exposure (29, 30). These phenotypically diverse subpopulations contribute to antibiotic tolerance and the prolonged treatment required for tuberculosis cure. However, detecting and quantifying these subpopulations *in vitro*, within infected macrophages (*in cellulo*) or tissue models (*in vivo*), has been technically challenging (31).

Previous approaches to exploring Mtb replication and physiological state have provided important layers of insight. The RNA-based RS ratio quantifies precursor rRNA relative to mature 23S rRNA as a molecular readout of ongoing rRNA synthesis (precursor rRNA [ETS1]/mature 23S rRNA), serving as a sensitive indicator of bacterial metabolic activity and drug response at the population level (32). In parallel, the SSB-GFP replication reporter enables direct visualization of active DNA replication forks through fluorescent foci, providing single-cell resolution of replication events (4). A Timer-Mtb strain complements these methods by adding a temporal dimension to single-bacterium analysis: through differential fluorophore maturation kinetics, it distinguishes recently synthesized from older proteins, allowing discrimination between actively growing, recently active, and quiescent bacilli within heterogeneous populations. Thus, while the RS ratio captures bulk transcriptional activity and SSB-GFP marks replication fork engagement, a Timer-based reporter integrates growth history at the individual cell level, offering a more refined framework to resolve Mtb activity states during infection or under drug pressure.

Mtb-Timer allows direct visualization of Mtb replication heterogeneity in real time, without the need for cell lysis or fixation. In human macrophage infection models, the Mtb-Timer approach provides a powerful tool to distinguish actively replicating from non-replicating bacilli, thus enabling the study of intracellular bacterial dynamics within their natural host environment. This is particularly valuable given that intracellular Mtb populations show marked heterogeneity in replication rate, redox balance, and metabolic activity (33, 34). Timer-based fluorescence enables continuous monitoring of these subpopulations. Moreover, the ratio metric nature of the reporter provides quantitative data that are independent of bacterial load. Future studies will reveal how Mtb-Timer responds to microenvironmental factors such as phagosome maturation, acidification, or exposure to host-derived stress signals.

The use of Mtb-Timer also offers key advantages for studying responses to antibiotics *in vitro* as well as in host environments. Unlike traditional viability assays that capture static endpoints, the Mtb-Timer reporter allows us to follow the temporal progression of bacterial responses to antibiotics. By measuring changes in green-to-red ratios, Mtb-Timer informs not only on whether replication is inhibited but also on whether the bacterial population remains metabolically active or transitions into a non-replicating state. This distinction is important for our understanding of the basis of antibiotic tolerance, as several studies have demonstrated that drug-exposed bacilli can persist in a non-dividing state (35, 36). *In vitro*, Mtb-Timer combined with the agarose pad system enables real-time, single-cell monitoring of Mtb responses to antibiotics under stable and reproducible conditions. Immobilizing bacteria minimizes movement and clumping, allowing precise quantification of fluorescence changes that reflect metabolic activity and growth arrest (37). This approach revealed phenotypic heterogeneity in drug-treated populations, and further studies using this reporter will contribute to our understanding of how heterogeneity drives phenotypic responses to antibiotics.

The Mtb-Timer reporter provides a unique combination of temporal resolution, quantitative precision, and compatibility with live cell imaging, making this reporter well suited for dissecting Mtb heterogeneity and drug response. By enabling real-time, single-cell level analysis of bacterial replication within complex environments, Mtb-Timer bridges a critical methodological gap in tuberculosis research. As the field continues to integrate dynamic imaging, systems biology, and single-cell analysis, Timer-based tools promise to become relevant for studying the fundamental biology of Mtb at the single-cell level. We anticipate that this tool will contribute to the development of anti-Mtb drug discovery pipelines.

## MATERIALS AND METHODS

### Generation of Mtb-Timer (Mtb H37Rv-Timer^Mtb^)

The DsRed1E5 sequence was taken from Terskikh et al. (13), codon optimized for Mtb, and synthesized as a gene fragment (GeneWiz, USA). This optimized Timer^Mtb^ sequence was cloned into the pDO23A plasmid (38) using BP clonase, and the Timer^Mtb^ sequence was subsequently cloned downstream of a *Mycobacterium* Strong Promoter (500 bp upstream of MMAR_5083) in an extrachromosomal replicating plasmid with a *Mycobacterium* replication origin and a hygromycin selection marker using Gateway cloning.

For transformation with Timer^Mtb^ plasmid, electrocompetent Mtb H37Rv cells were generated by expanding a starter culture in the exponential phase into a roller bottle containing 100 mL sterile 7H9 medium (BD Difco, 271310) supplemented with 10% ADC (BD Difco, 212352) and 0.05% Tween 80 (Sigma Aldrich, 9005-65-6) and incubated at 37°C until OD_600nm_ reaches ~1.0. To increase cell wall permeability, the culture was supplemented with 2 M glycine (Sigma-Aldrich, 50046) and incubated for an additional 20 h. Cells were harvested by centrifugation at 3,000 rpm for 5 min and repeatedly washed with sterile 10% glycerol (Fisher Scientific, 10021083), gradually reducing the wash volume: 25 mL/12.5 mL/6.25 mL. After the final wash, the pellet was resuspended in 5 mL of 10% glycerol, yielding an electrocompetent cell suspension used immediately for transformation. For electroporation, 1 µg of salt-free Timer^Mtb^ plasmid DNA was mixed with 100 µL of electrocompetent cells in 2 mm electroporation cuvettes (Bio-Rad, 1652086). Electroporation was carried out using a Gene Pulser Xcell Electroporation System (Bio-Rad, 1652660) with the following parameters: 2,500 V, 1,000 Ω, and 25 μF. Following pulse delivery, 1 mL of fresh pre-warmed 7H9 + 10% ADC + 0.05% Tween 80 recovery medium was added directly to the cuvettes, and the cultures were incubated overnight at 37°C to allow recovery. The recovered cells were then plated onto selective 7H11 agar (BD Difco, L12203) containing 50 µg/mL of hygromycin (Gibco, 10687010) and incubated at 37°C for 3 weeks. Plates were inspected periodically to confirm transformant growth. Three positive Mtb H37Rv-Timer^Mtb^ colonies were picked and grown in 7H9 medium containing hygromycin, and aliquoted stocks were prepared and stored at −80°C for future use. For brevity, the Mtb H37Rv-Timer^Mtb^ strain is referred to throughout the manuscript as Mtb-Timer.

Next, the Mtb-Timer strain (Fig. 1A) was tested for the presence of phthiocerol dimycocerosates (PDIMs) by thin-layer chromatography (TLC) after lipid extraction: heat-killed pellets were washed with phosphate-buffered saline (PBS) and extracted with methanol (Sigma Aldrich, 322415)/chloroform (Fisher Scientific, 10293850) (2:1) overnight, followed by a second extraction in methanol/chloroform (1:1). Combined extracts were dried, resuspended in chloroform, and 30 µL was applied to silica TLC plates (Merck, 1055700001). Purified PDIM (BEI Resources, NR-20328) was included as a positive control. Plates were developed twice in petroleum ether (Sigma-Aldrich, 320447)/ethyl acetate (Fisher Scientific, 10386320) (98:2), sprayed with 5% phosphomolybdic acid (Sigma-Aldrich, 221856), and heated to visualize the PDIM bands.

### Agarose pad-based *in vitro* assay for single-cell Mtb-Timer live imaging

Single-cell suspensions of Mtb-Timer were prepared for imaging using an adapted agarose pad protocol. Ultra-low melting point (ULMP) agarose (Lonza, 50302) was prepared at 1.5% in sterile deionized water, melted at 95°C, and kept in 2 mL aliquots. Bacterial cultures were pelleted by centrifugation at 3,000 rpm for 5 min, subjected to 1 min of glass bead beating vortexing, followed by 1 min of vigorous shaking to facilitate bacterial dispersion, and resuspended in 5 mL of 2× concentrated 7H9 media. The optical density of each suspension was adjusted to OD_600_ = 2, and cells were filtered through 1.2 μm syringe filters (Sartorius, FC124) to remove clumps. For agarose pad preparation, 750 μL of melted ULMP agarose was mixed with 750 μL of bacterial suspension in 2× 7H9, and 100 μL of the mixture was dispensed into each well of a 96-well plate (Revvity, 6055600). Plates were centrifuged at 3,000 × *g* for 30 min to sediment bacteria at the bottom of the wells, followed by cold centrifugation at 4°C and 1,500 × *g* for 20 min to solidify the agarose pads. Antibiotic treatments were applied directly onto the solidified pads by adding 50 µL of suspension to diffuse. Plates were incubated overnight at 37°C, and imaging was performed 24 h after antibiotic exposure using an Opera Phenix Microscope (Revvity, HH14002001) with a 63 × 1.15 NA water immersion objective. Image acquisition and quantitative comparison of conditions were performed using the Harmony high-content imaging software (Revvity, HH17000019), which allowed segmentation of individual bacteria and quantification of the bacterial area in green, intermediate (orange), and red fluorescence states, based on the green to red ratio (Fig. S1).

### Isolation and differentiation of human monocyte-derived macrophages

Human monocyte-derived macrophages (HMDMs) were obtained from leukocyte cones provided by the UK National Health Service. Peripheral blood leukocyte cones (~10 mL) were drained into Falcon tubes and diluted with 20 mL phosphate-buffered saline containing 2 mM EDTA (autoMACS buffer) (Miltenyi Biotec, 130-091-222). The diluted blood was layered over Ficoll-Paque PLUS (Merck, GE17-5442-02) (5.6 mL per sample) and centrifuged at 300 × *g* for 1 h at room temperature, with slow acceleration and no brake. The mononuclear cell layer at the interface was collected, washed twice with autoMACS buffer, and subjected to red blood cell lysis using 10 mL RBC lysis buffer (Sigma-Aldrich, R7757) for 20 min at 4°C. Cells were then washed again in autoMACS buffer, resuspended in ice-cold autoMACS + 5% BSA (Cell Signaling Technology, 9998S), and counted using a LUNA FX7 (Logos Biosystems) cell counter. Cell suspensions were centrifuged (300 × *g* for 10 min at 4°C) and resuspended in autoMACS + 5% BSA. CD14 MicroBeads (Miltenyi, 130-050-201) were added and incubated for 15 min at 4°C. After washing, labeled cells were separated using MACS LS columns (Miltenyi, 130-091-051) placed in a magnetic field according to the manufacturer’s protocol. Columns were washed three times with autoMACS + 5% BSA, and CD14^+^ monocytes were eluted by removing the column from the magnet and flushing it with MACS buffer. The purified monocytes were pelleted and resuspended in pre-warmed RPMI 1640 supplemented with 10% FBS (Thermo Fisher Scientific, 72400054).

Purified monocytes were plated at 1.2 × 10^6^ cells/mL in 9 cm petri dishes containing RPMI + 10% FBS supplemented with 50 ng/mL of human granulocyte-macrophage colony-stimulating factor (GM-CSF) for differentiation and maintained at 37°C and 5% CO_2_. After 3 days, plates were washed with PBS, and additional fresh GM-CSF-supplemented medium was added to each dish. After another 3 days, cells exhibited characteristic macrophage morphology. For detachment, the medium was removed, cells were washed once with ice-cold PBS-EDTA, and incubated with 5 mL of PBS-EDTA for 15–20 min at 4°C. Loosened cells were scraped and collected into 50 mL tubes, washed with RPMI + FCS, and pelleted (350 × *g* for 7 min at room temperature). Macrophages were resuspended in fresh RPMI + 10% FBS, counted using a LUNA FX7 cell counter, and adjusted to ~60,000 cells per well for plating into flat-bottom black 96-well plates (Revvity, 6055600).

### Mtb infection of human primary macrophages

Mtb-Timer was grown in Middlebrook 7H9 + 10% ADC + 0.05% Tween 80 supplemented with 50 μg/mL hygromycin to maintain plasmid selection. Cultures were incubated at 37°C in a rotary wheel to ensure proper aeration and homogeneous growth until exponential growth was achieved (OD_600nm_ = 0.6–1). To prepare Mtb single-cell suspensions for infection, cultures were centrifuged at 3,000 rpm for 5 min. The supernatant was discarded by decanting, and the bacterial pellet was washed with 15 mL of PBS. This wash step was repeated using 15 mL of PBS to ensure full removal of the original mycobacterial culture media. After removing the supernatant, sterile 2 mm glass beads were added to the pellet and vortexed to disrupt mycobacterial clumps. Pre-warmed complete RPMI + 10% FBS medium was then added to the tube. The resulting suspension was transferred to clean 15 mL Falcon tubes and centrifuged at 1,200 rpm for 5 min to pellet remaining clumps. The supernatant, containing the single-cell bacterial suspension, was retained. Optical density at 600 nm was measured to estimate bacterial concentration, with the assumption that an OD_600nm_ = 1 corresponds to approximately 10^8^ CFU/mL of Mtb. The suspension was then diluted to achieve an MOI of 1:1 (bacterium: macrophage).

Differentiated HMDM cells, pre-seeded in 96-well plates, were prepared for infection by removing the culture medium using a multichannel pipette. Each well then received 50 μL of the prepared Mtb single-cell suspension. Plates were incubated at 37°C with 5% CO_2_ for 2 h to allow bacterial uptake. Following infection, the medium was carefully removed, cells were washed with PBS, and fresh, pre-warmed RPMI supplemented with 10% FBS was added. Infected macrophages were further incubated for 24 h under the same conditions prior to treatment. Antibiotic treatments were performed using low (1/16 EC_99_), medium (1/4 EC_99_), and high (EC_99_) concentrations: INH (Merck, I3377), 0.04, 0.16, and 0.64 µg/mL; RIF (LKT Labs, R3220), 0.125, 0.5, and 2 µg/mL; EMB (Sigma-Aldrich, E4630), 0.2, 0.8, and 3.2 µg/mL; PZA (Sigma-Aldrich, P7136), 25, 100, and 400 µg/mL.

### Live and fixed cell imaging of Mtb-Timer-infected human macrophages

Infected HMDMs were either fixed at defined time points or monitored live over time using high-content fluorescence microscopy. For fixed imaging, macrophages were fixed at 2, 24, 48, 72, and 96 h post-infection with 4% paraformaldehyde (PFA) (EMS, 15710-S) overnight at 4°C. Following fixation, plates were washed twice with PBS and incubated with 100 μL of 1× ammonium chloride (NH_4_Cl) for 10 min at room temperature to quench the autofluorescence from PFA fixation. After washing, nuclei were stained with DAPI (1:10,000 dilution in PBS) for 10 min in the dark. DAPI was then removed, wells were washed once with PBS, and plates were sealed with parafilm and stored at 4°C in the dark until imaging in the Opera Phenix microscope.

Live-cell imaging was performed 24 h post-infection and after antibiotic treatment for up to 96 h post-infection using the Opera Phenix High-Content Screening Microscope equipped with environmental control for temperature (37°C) and CO_2_ (5%). Images were acquired every 1.5 h using a 40× water immersion objective, with sequential excitation at 488 nm (green Timer emission) and 561 nm (red Timer emission). For fixed samples, identical imaging parameters were used to ensure comparability between data sets. Image processing and quantitative analysis were conducted using Harmony software (PerkinElmer). Segmentation masks were applied to identify macrophage cytoplasm and intracellular Mtb objects. Bacterial area and mean green-to-red fluorescence intensity ratios were extracted at the population level. Data were used to correlate Mtb-Timer fluorescence shift with bacterial replication (area) and antibiotic-induced growth inhibition. Representative fluorescence images were generated using maximum intensity projections of three confocal z-stacks.

### Flow cytometry analysis of *in vitro* Mtb cultures

For the analysis of Mtb in *in vitro* cultures, at 24 and 48 h post-antibiotic treatment, Mtb cultures were harvested, washed with PBS, and resuspended in 4% PFA in PBS for overnight fixation at 4°C. For the analysis of intracellular Mtb in THP-1, at 72 h post-infection, infected THP-1 were washed with PBS and lysed in 0.05% Triton-X-100 in H_2_O at 37°C for 5 min to release intracellular Mtb. Released Mtb was fixed overnight in 4% PFA in PBS at 4°C. The fixed Mtb were washed with PBS and analyzed using a BD LSRFortessa Cell Analyzer (BD Biosciences). Data were analyzed using FlowJo software (FlowJo, LLC, v10.10.1). At least 10,000 events per condition were recorded using an optimized gating strategy (Fig. S2B).

### Flow cytometry analysis of Mtb-Timer-infected THP-1

THP-1 (TIB-202) human monocyte cell line was obtained from American Type Culture Collection and routinely cultured in RPMI-1640 + 10% FBS at 37°C with 5% CO_2_ and 100% humidity. Three days prior to Mtb infection, THP-1 were differentiated in RPMI-1640 + 10% FBS + 10 µg/mL phorbol 12-myristate 13-acetate (PMA) (Merck, P1585) for 24 h and rested in medium without PMA until infection. THP-1 were infected with Mtb and treated with antibiotics following the same workflow to that of HMDMs. At 72 h post-infection, THP-1 were washed once with PBS, detached using TrypLE (Gibco, 12604013) with cell scraping, and fixed in 4% PFA in PBS overnight at 4°C. The fixed cells were washed with PBS and analyzed using a BD LSRFortessa Cell Analyzer (BD Biosciences), and the data were analyzed using FlowJo software (FlowJo, LLC, v10.10.1). At least 10,000 events per condition were recorded using an optimized gating strategy (Fig. S2C).

### Statistical analysis

Statistical analyses were performed using GraphPad Prism v10.6.1. *P*-values were calculated using two-way ANOVA, followed by Dunnett’s multiple comparisons test, comparing each antibiotic dose (low [1/16 EC_99_], medium [1/4 EC_99_], and high [EC_99_]) to the control condition for both Mtb-Timer area and green-to-red ratio measurements over time. Significance levels were defined as follows: ns, not significant (*P* > 0.05); *P ≤ 0.05; **P ≤ 0.01; ***P ≤ 0.001; and ****P ≤ 0.0001. Figures were prepared using Adobe Illustrator 29.8.1.

## SUPPLEMENTAL MATERIAL

Supplemental Figures msystems.01796-25-s0001.pdf Fig. S1 and S2.

ASM does not own the copyrights to Supplemental Material that may be linked to, or accessed through, an article. The authors have granted ASM a non-exclusive, world-wide license to publish the Supplemental Material files. Please contact the corresponding author directly for reuse.
