# Supplementary material for: Mtb-Timer: a fluorescent reporter to visualize Mycobacterium tuberculosis replication and antibiotic responses
Source: mSystems. 2026 May 19;11(6):e01796-25. doi: 10.1128/msystems.01796-25 (PMC13288934; doi:10.1128/msystems.01796-25)
Supplement: Supplemental Figures — Fig. S1 and S2. [file msystems.01796-25-s0001.pdf]

**Supplemental material**

Víctor Campo-Pérez et al.

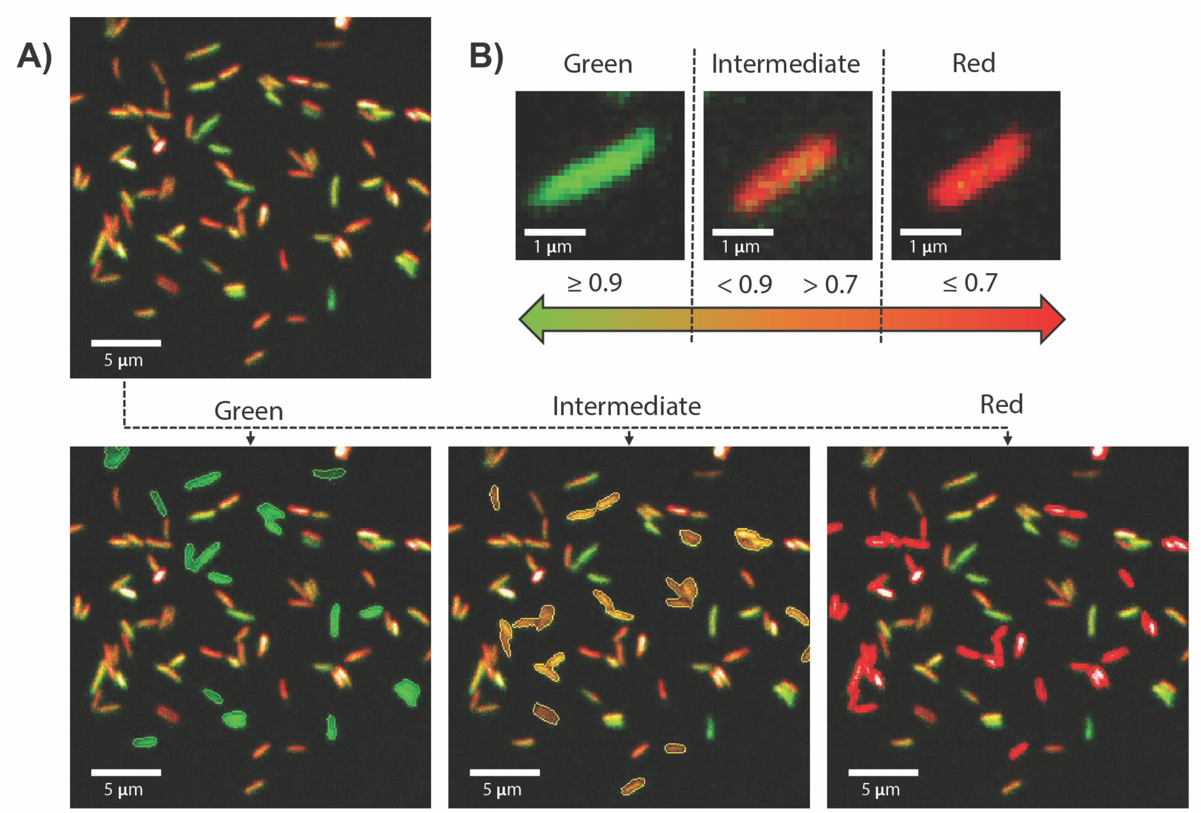


**Supplementary Figure 1. Segmentation of Mtb-Timer bacilli based on green-to-red fluorescence ratios. A)** Field of view containing fluorescence heterogeneous bacilli. Bacteria were segmented based on their green-to-red fluorescence ratio: ≥ 0.9 (green, actively replicating), < 0.9 and > 0.7 (orange/yellow, intermediate state), and ≤ 0.7 (red). **B)** Representative images of single bacteria appearing predominantly in each of the defined subpopulations.

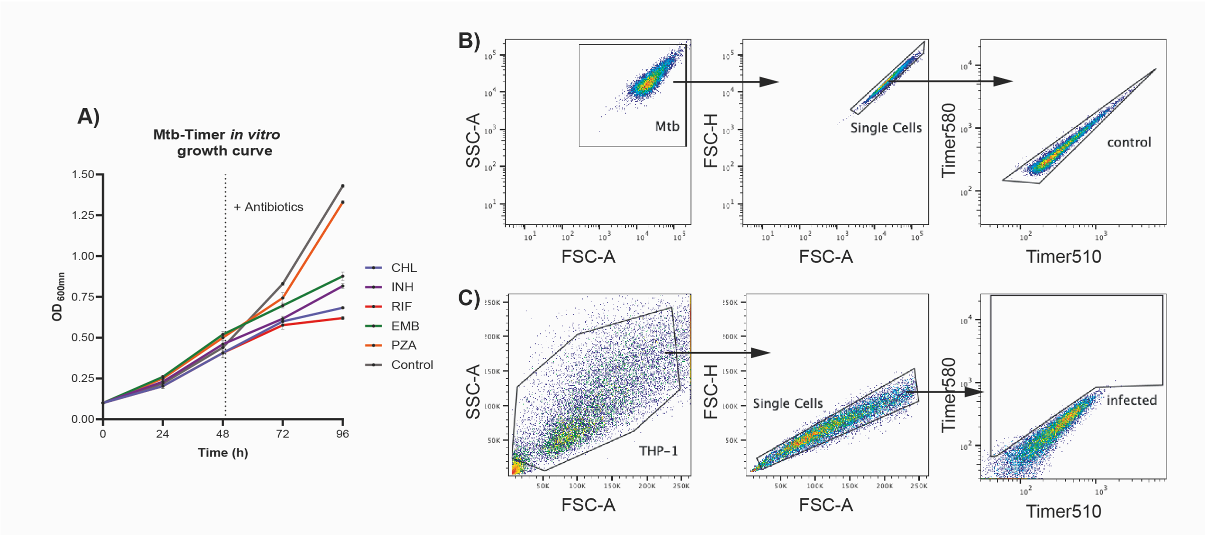


**Supplementary Figure 2. Gating strategy and growth curve of Mtb-Timer. A)** Growth curve of Mtb-Timer *in vitro* cultures used for flow cytometry experiments. **B)** Flow cytometry plots showing the gating strategy of Mtb. **C)** Flow cytometry plots showing the gating strategy of THP-1 cells.
